# Case Report: Successful management of refractory palmoplantar pustulosis with upadacitinib

**DOI:** 10.3389/fimmu.2025.1476584

**Published:** 2025-03-10

**Authors:** Boyun Yang, Hanxiao Yu, Wo Yao, Huiying Wang

**Affiliations:** ^1^ Department of Allergy, Second Affiliated Hospital of Zhejiang University School of Medicine, Hangzhou, Zhejiang, China; ^2^ Clinical Research Center, Second Affiliated Hospital of Zhejiang University School of Medicine, Hangzhou, Zhejiang, China

**Keywords:** palmoplantar pustulosis, anti-IgE, upadacitinib, Janus kinase inhibitors, cytokines

## Abstract

Palmoplantar Pustulosis (PPP) is a rare chronic skin disorder characterized by recurrent sterile pustules on palms and soles, leading to significant pain and functional impairment. Treatments include topical medications, phototherapy, systemic treatments, and biologics, but nonconclusive strategy exists. Here we report a case of a 66-year-old Chinese woman who developed refractory PPP after COVID-19 vaccination, characterized by painful, itchy pustules on her hands and feet. Initial treatments such as topical corticosteroids, calcipotriol, methotrexate, and cyclosporine were ineffective. Due to potential hypersensitivity reactions post-vaccination and elevated Immunoglobulin (Ig)E levels, anti-IgE therapy was administrated. Omalizumab treatment resulted some improvement, but noticeable symptoms persisted. Upon switching to upadacitinib, the patient experienced rapid and complete resolution of pustules and desquamation, with continued symptom control and no severe adverse reactions over a year. Throughout the treatment, clinical symptoms and the patient’s quality of life were assessed using the Palmoplantar Pustular Psoriasis Area and Severity Index (PPP ASI), the Palmoplantar Pustulosis Physician Global Assessment (PPP PGA), and the Dermatology Life Quality Index (DLQI). Serum IgE and food-specific (FS)-IgG4 levels were monitored. Additionally, reductions in cytokine levels (interleukin (IL)-4, IL-13, IL-25, IL-33, and tumor necrosis factor (TNF)-α) were observed after upadacitinib treatment. This case highlights the potential of upadacitinib, as an effective treatment for PPP, emphasizing the need for further research into targeted therapies addressing multiple signaling pathways involved in PPP’s pathogenesis.

## Introduction

Palmoplantar Pustulosis (PPP) is a chronic, recalcitrant inflammatory skin disease marked by recurrent sterile pustules on the palms and soles, leading to disproportionate pain, pruritus, and functional impairment (1, 2). PPP is rare, with a higher prevalence in Japan (0.12%) compared to the United States and Europe (0.001% to 0.08%) (3–5). It predominantly affects individuals aged 40 to 58 years (6), with a notable inclination towards women (7). There is ongoing controversy regarding its classification, with uncertainty about whether PPP represents a subtype of palmoplantar psoriasis, a localized form of pustular psoriasis, or a distinct condition (8). Current therapeutic strategies involve the use of topical medications (e.g., corticosteroids and active vitamin D3) (4, 9), phototherapy (PUVA) (10), oral systemic treatments (e.g., acitretin or etretinate, cyclosporine, methotrexate, tetracyclines, apremilast) (9, 11), and biologic injectable treatments (12, 13).

Here we reported a case of PPP initialed after COVID-19 vaccination. After the failure of treatment with multiple conventional medications, partial relief with omalizumab, the patient achieved complete remission with upadacitinib therapy. In this study, we monitored the clinical efficacy and adverse reactions of omalizumab and upadacitinib treatments for PPP. Additionally, we regularly measured serum Immunoglobulin (Ig)E antibody levels and various cytokines including interleukin (IL)-4, IL-13, IL-25, IL-33 and tumor necrosis factor (TNF)-α to explore treatment strategies for PPP.

## Case report

**Figure 1:**
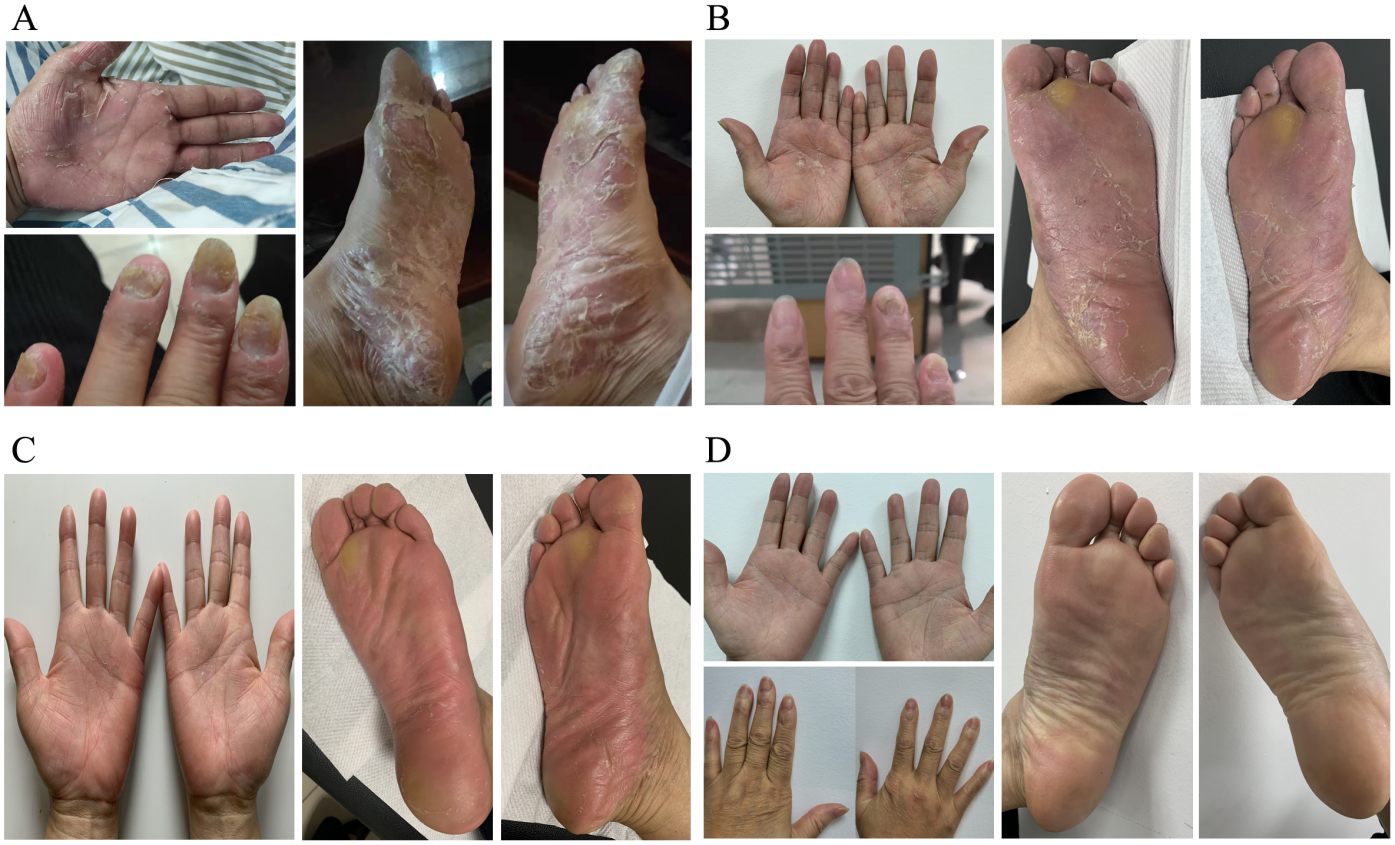
The clinical images of Palmoplantar Pustulosis Psoriasis (PPP) before and after treatment. **(A)**Before treatment: lesions on the left palm, left fingernails, and soles of both feet. **(B)**After 6 cycles of treatment with omalizumab, manifestations of palmoplantar lesions. **(C)**After 1 month of treatment with upadacitinib, manifestations of palmoplantar lesions. **(D)**After 3 months of treatment with upadacitinib, manifestations of palmoplantar lesions and fingernail abnormalities in both hands.

After receiving the COVID-19 vaccine, a 66-year-old Chinese female patient encountered a sudden onset of erythematous skin rash accompanied by painful and itchy pustules on her hands and feet, persisting for five months. Following this, the pustules ruptured, leading to the development of thickened, excessively keratinized scales that peeled off from the affected red areas. At the same time, she observed yellowing and increased fragility in the fingernails on both hands. The physical examination revealed blistering with erythema and desquamation on the palms of both hands and the soles of both feet. The fingernails of both hands were yellowish-gray, accompanied by nail plate fragmentation and subungual blisters (**Figure 1A**). Immunological test results indicated a total IgE level of 2979.88 IU/ml, significantly higher than the normal range (<100 IU/ml). Specific IgE tests were all negative (normal <0.35 IU/ml), while food-specific (FS)-IgG4 shows an elevated reaction to eggs at 878.8 U/ml (normal <250 U/ml). Routine blood tests, liver and kidney function, and coagulation function all showed no apparent abnormalities. No family history of psoriasis vulgaris or plaque psoriasis was noted, neither was smoking experience. During her treatment, local therapies, including potent corticosteroids, topical calcipotriol, as well as systemic medications such as methotrexate (5 mg twice a week), tripterygium wilfordii tablets (20 mg twice daily), paeoniflorin (600 mg twice daily), compound glycyrrhizin tablets (50 mg three times daily), and loratadine tablets (10 mg once daily), all ended in failure. Subsequently, she commenced combined therapy with tripterygium wilfordii tablets and anti-IgE treatment.

**Figure 2:**
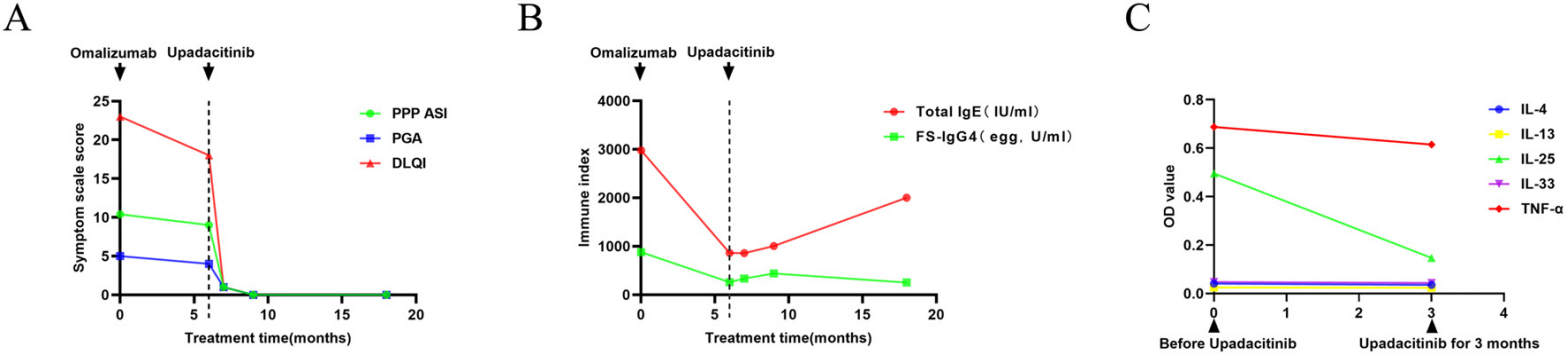
Curve of index changes during treatments. **(A)** The changes in the Palmoplantar Pustulosis Psoriasis Area and Severity Index (PPP PASI), Patient Global Assessment (PGA), and Dermatology Life Quality Index (DLQI) during the treatment process. **(B)** The changes in serum total immunoglobulin E (IgE) and food-specific (FS)- IgG4 (egg) during the treatment process. **(C)** The changes in cytokines interleukin (IL)-4, IL-13, IL-25, IL-33, and tumor necrosis factor-alpha (TNF-α) before and after treatment with upadacitinib.

She was then on the administration of omalizumab subcutaneously at 300mg/day at a four-week interval. After 4 cycles of omalizumab treatment, oral tripterygium wilfordii tablets was tapered gradually and finally discontinued, with only topical calcipotriol used in conjunction. Following a total of 6 cycles of omalizumab, the patient’s symptoms improved to a certain extent, but there was still noticeable erythema, with pustules erupting. Desquamation was evident, and nails continued to detach. The skin on the palms and soles felt tight, accompanied by a sense of pain, especially on the soles. On severe conditions, there was oozing and an unpleasant odor (**Figure 1B**). Additionally, upon re-evaluation of half a year into treatment, allergy testing indicated a total IgE level of 863 IU/ml (**Figure 2B**).

With the introduction of a highly selective Janus kinase (JAK) 1 inhibitor, and upon obtaining the patient’s consent, we adjusted the treatment plan, prescribing upadacitinib at 15mg orally once daily. The skin on the palms and soles showed significant improvement within one week of medication. By the end of the first month, pustules and desquamation had completely resolved whilst erythema persisted (**Figure 1C**). The symptoms of pain, oozing, or odor almost disappeared completely. Three months later, her skin and nails returned to a normal state without erythema anymore (**Figure 1D**). Upadacitinib tapered gradually then with a decrease of one tablet per week in the fourth month, two tablets per week in the fifth month, and three tablets per week in the sixth month, till 15mg every five days as maintaining dose for a year without symptoms’ recurrence. The patient tolerated upadacitinib well, despite occasional scattered acne on the face. No revere infections or other cardiovascular diseases were reported. Regular monitoring of blood routine, liver and kidney function, coagulation function, lipid profile, hepatitis markers, and T-SPOT test for tuberculosis during the medication period revealed no significant abnormalities. Furthermore, the total IgE level increased to 2000 IU/ml, whereas the FS-IgG4 antibodies to eggs gradually declined as a result of ongoing dietary elimination and had since normalized (**Figure 2B**.). Throughout the treatment process, we assessed the severity of skin lesions and their impact on the quality of life of the patient using the Palmoplantar Pustular Psoriasis Area and Severity Index (PPP ASI), the Palmoplantar Pustulosis Physician Global Assessment (PPP PGA), and the Dermatology Life Quality Index (DLQI) (**Figure 2A**). Simultaneously, we assessed various cytokine levels before and one year after upadacitinib treatment. We observed a reduction in IL-4, IL-13, IL-25, IL-33, and TNF-α levels after upadacitinib therapy, with IL-25 showing the most significant decrease (**Figure 2C**). Unfortunately, we lacked serum samples from before omalizumab treatment. The treatment regimen and follow-up are still ongoing. Subsequent steps will involve further medication tapering, and possibly discontinuation, while closely monitoring clinical symptoms and adverse reactions.

## Discussion

In this report, we present a case of refractory PPP successfully treated with upadacitinib after multiple prior medications proved ineffective. Meanwhile, we observed the symptom improvement, cytokines change, trying to explore the potential mechanism of the treatment of upadacitinib.

The pathogenesis of PPP is complex, involving a combination of environmental triggers, genetic predisposition, and both innate and adaptive immune responses. In the case of this patient, she was previously healthy without smoking experience and family history. Metal allergies and emotional issues were also excluded. The symptoms began after a COVID-19 vaccination, which indicated a possible trigger.

Elevated IgE levels are found in some PPP patients, especially those with an allergic component (14). IgE can activate immune cells like mast cells and dendritic cells to release cytokines (e.g., IL-17, IL-22, TNF-α), contributing to inflammation in psoriasis (15). Although omalizumab has not been used for PPP before, we started anti-IgE therapy due to the risk of hypersensitivity reactions after vaccination and the patient’s high IgE levels. In patients with concomitant inflammatory dermatoses, such as urticaria and psoriasis, the combination of omalizumab with secukinumab or tildrakizumab has demonstrated promising therapeutic outcomes. Omalizumab was chosen for its anti-IgE and anti-inflammatory effects, which may help modulate the immune response in PPP (16, 17). As Gadir suggested, it may increase Treg cell activity, restoring balance between T-help (h)17 and Treg cells, and promoting immune tolerance while reducing autoimmunity (18). Although the treatment showed some improvement, it did not achieve complete control of the condition. As a result, we promptly switched to JAK inhibitors to better manage the patient’s symptoms. PPP is closely linked to psoriasis, prompting clinical studies to assess the effectiveness of biologic treatments approved for psoriasis or psoriatic arthritis in managing PPP. In the immune mechanisms of PPP, elevated levels of multiple cytokines are involved, among which the IL-23/IL-17 inflammatory pathway has recently been considered to play an important role (19–21). However, in randomized controlled trials, biologics targeting this typical pathway did not demonstrate the same impressive efficacy as seen in plaque psoriasis (12, 22). Despite robust clinical studies and case reports, there is currently no conclusive treatment for PPP, nor are published management guidelines. Due to PPP involving multiple signaling pathways, JAK inhibitors may indeed be an ideal treatment option. Tofacitinib, baricitinib, and upadacitinib inhibit JAK1/3, JAK1/2, and JAK1, respectively, demonstrating significant efficacy and good tolerability in clinical cases of PPP (6, 23, 24). Recent case series have retrospectively evaluated the short-term clinical efficacy and safety of upadacitinib in the treatment of PPP, providing further evidence that upadacitinib may serve as an effective and well-tolerated therapeutic option for this condition (25–27). In our patient, upadacitinib demonstrated strong efficacy and good tolerability. After one year of treatment, the dosage was reduced to 15 mg every 5 days. This suggests that long-term upadacitinib can maintain symptom remission and lower recurrence rates. We also observed a decrease in multiple cytokine levels after treatment, particularly in the Th2 and Th17 pathways, suggesting that single-pathway therapies may not fully address PPP. As previous studies have shown, PPP involves a complex immune response, with Th2 activation in the skin and a shift of circulating T cells towards a Th17 phenotype (27). Similarly, other case reports show that patients poorly responded to multiple biologics but responded well to upadacitinib (28, 29).

In conclusion, upadacitinib proved to be an effective treatment for refractory PPP, providing rapid and strong symptom control in the short term. Long-term treatment not only reduced symptom recurrence but also enabled medication discontinuation. The patient tolerated the treatment well, highlighting upadacitinib as a highly promising therapeutic option.

## Data Availability

The original contributions presented in the study are included in the article/supplementary material. Further inquiries can be directed to the corresponding author.
